# Enhanced Upper Extremity Functions with a Single Session of Action-Observation-Execution and Accelerated Skill Acquisition Program in Subacute Stroke

**DOI:** 10.1155/2018/1490692

**Published:** 2018-06-12

**Authors:** Shambhu Prasad Adhikari, Jarugool Tretriluxana, Pakaratee Chaiyawat, Chutima Jalayondeja

**Affiliations:** ^1^Faculty of Physical Therapy, Mahidol University, Thailand; ^2^Department of Physiotherapy, Kathmandu University School of Medical Sciences, Nepal

## Abstract

**Background:**

Action-observation-execution (AOE) primes physical training. We examined the immediate effect of AOE with accelerated skill acquisition program (ASAP) on dexterity in subacute stroke.

**Methods:**

Twelve individuals from 1 to 6 months after stroke were allocated into two groups by matching age and side of stroke. After AOE of 30 minutes, the experimental group received ASAP for 60 minutes whereas the control group received dose-equivalent usual care. The movement time (MT) and functional ability (FA) of hand items of the Wolf motor function test (WMFT), hand functions and global recovery of stroke impact scale (SIS), and intrinsic motivation items of stroke rehabilitation motivation scale were assessed at baseline, after training, and during one-week follow-up. Data were analyzed within and between the groups.

**Results:**

AOE significantly decreased MT of flipping cards of WMFT and hand functions of SIS. Total MT was markedly reduced. AOE with ASAP demonstrated significant group-by-time interactions on MT of lifting pencil of WMFT, total MT, and global recovery. Grip strength, FA, and hand functions were significantly improved only in the experimental group. Both groups improved motivation significantly.

**Conclusions:**

The AOE with ASAP enhanced dexterity, which persisted for at least a week. This intervention might improve dexterity in subacute stroke.

**Trial Registration Number:**

This trial is registered with TCTR20161007001.

## 1. Background

Stroke is a leading cause of long-term disability globally. [1] About 50–60% stroke survivors are left with residual impairments [2]. Middle cerebral artery syndrome is the most common type which impairs upper extremity (UE) more significantly than lower extremity [3]. The UE recovery is poorer compared with lower extremity recovery and is a challenge in rehabilitation [4]. Even mild UE impairments after stroke significantly limit daily activities and negatively impact quality of life [5]. Evidence shows increasing attention to mirror neuron system-based interventions for functional training in stroke [6–10].

Action-observation (AO) is a systematic observation by which the brain matches an observed action to its motor counterparts via mirror neuron system (MNS) [6]. It induces neuronal reorganization similar to that induced by physical training [11–13]. AO is reinforced by the consecutive execution of the observed action termed action-observation-execution (AOE) [14]. AOE involves larger set of brain regions (dorsolateral and dorsal premotor cortex, presupplementary motor area, superior parietal, and superior temporal lobules) and greater neuronal reorganization with increased mirror neurons' activity compared with AO [6, 8]. The AOE induces neural plasticity that was demonstrated through functional magnetic resonance imaging (fMRI) and behavioral measures in chronic [15, 16] and acute [6, 7] stroke. Although, the subacute [17] phase of stroke is a golden period of recovery [18] and a stage of neuronal reorganization [19], the effect of AOE in subacute stroke has not been studied. In addition, motivation is a key element for engaging participants in AOE and is a contributor of continued choice, effort, and persistence to use paretic limbs [19–21]. Motivation helps in reduction of self-imposed participation restriction, increases participation level, improves self-confidence, and reinforces the linkage between skills, capacity, and intrinsic drive to support acquisition of skills [22] through modulation of the mirror neuron system [23]. Although, different strategies have been applied to motivate participants in previous AOE related studies, [4, 6, 8, 16] a protocol with structured motivation component was lacking.

Motor priming, when delivered prior to or in conjunction with the primary intervention facilitates motor learning and induces neuroplasticity with improvements in motor performance [24]. The AO or AOE in combination with physical practice has improved upper extremity motor functions in stroke rehabilitation more effectively than physical practice alone [8, 14, 25]. It is because the combination optimizes the plastic changes induced by physical practice and results in remarkable as well as long-term performance gain [26]. There is no evidence of any contemporary motor training techniques primed with AOE for UE training.

The accelerated skill acquisition program (ASAP) is an evidence-based contemporary motor training intervention that integrates motivation component together with skills and capacity [22]. In this patient-centered intervention, the acquisition of skilled movements is achieved through task-oriented training, capacity is increased through impairment reduction, and self-confidence is built through patient's active involvement in task selection, problem solving, and decision-making [21, 22]. Faster performance, improved quality of movement [27, 28], and better functional improvement compared with the usual care [27] have been demonstrated with ASAP. Therefore, the neuronal plasticity could be further enhanced, if ASAP is primed with AOE, through the activation of common neural substrate [13, 29]. The priming effect of AOE on conventional physiotherapy is evident in chronic [4, 15, 16] and early stages, [6–8] but not in subacute stroke. Moreover, there is no evidence on ASAP primed with AOE. Therefore, we examined the immediate effect of AOE combined with ASAP on hand function in subacute stroke.

## 2. Methods

### 2.1. Participants

Twelve individuals between 18 to 75 years old with unilateral stroke, middle cerebral artery syndrome, a period of 1 to 6 months after stroke [18], mild-to-moderate impairments (motor and coordination scores 31 to 55 out of 66) on Fugl-Meyer assessment (FMA) of UE [30], at least a score of “1” on hand mass flexion/extension [31], no cognitive impairments (mini-mental state examination ≥ 24/30) [32], and normal or corrected vision, National Institutes of Health Stroke Scale (NIHSS) = 0 [33, 34], and who were right-handed prior to stroke (Edinburgh handedness inventory: score ≥ 8/10) [35] were included. The exclusion criteria were severe pain on UE (FMA pain = 0 on ≥ 3/5 joints), visual neglect (NIHSS-neglect ≥ 1) [33, 34], severely restricted shoulder and elbow movement (FMA passive joint motion = 0), severe to total sensory loss in UE (NIHSS-sensory = 2) [33], and inability to sit independently for at least one hour. Ethical approval was received from Institutional Review Committee, Kathmandu University School of Medical Sciences, Nepal (57/15). Written informed consent was obtained from all participants prior to data collection.

### 2.2. Research Design and Procedure

**Figure 1:**
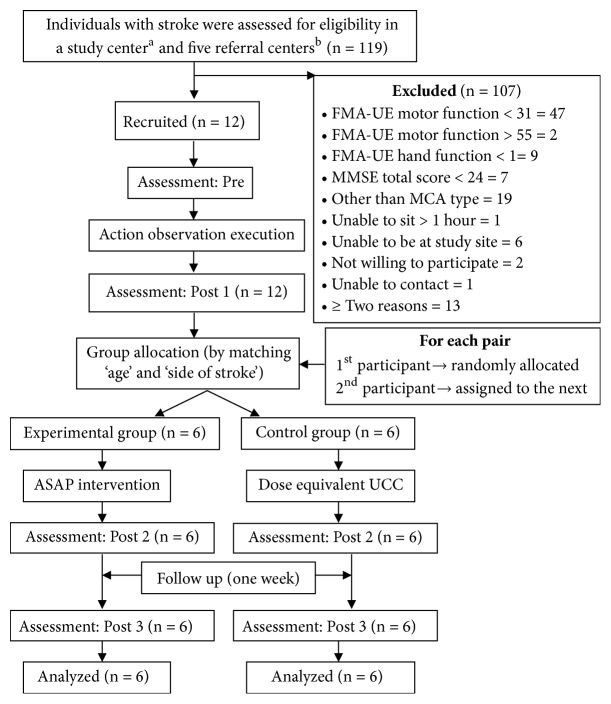
Consort flow diagram. ASAP: accelerated skill acquisition program, UCC: usual and customary care, n: number of participants, FMA-UE: Fugl-Meyer assessment-upper extremity, MMSE: mini-mental state examination, MCA: middle cerebral artery; Kathmandu University Hospital ^a^, Kathmandu Medical College Hospital ^b^, Annapurna Neurological Institute and Allied Sciences ^b^, Spark Health Home Hospital ^b^, Sahara Care Hospital ^b^ and Wellness Hospital ^b^ (first three are hospitals and last three are rehabilitation centers).

Individuals with stroke were screened in six different centers (five referral and one study center). However, recruitment was done only at the study center. The assessor and participants were blinded to group allocation. Each participant was intervened and assessed at different points of time. Assessor was blinded both for the intervention arm and for the hypothesis and was responsible only for the assessment. It was because we aimed to minimize the bias or error that could be from participants or assessor intentionally or unintentionally. Participants were allocated into two groups by matching age (± 5 years) and side of stroke (Figure 1). From a unit of each pair, first participant was randomly allocated to either experimental or control group. Its matched pair was then automatically assigned to another group. Evaluation was done before and immediately after each intervention and one week later [28, 36, 37] (Figure 1). The assessor was responsible only for the assessment. The research assistant (other than the assessor) was responsible for group allocation and intervention administration (AOE to all the participants, ASAP to only the experimental group) for which she was trained and standardized. Registered physiotherapists (other than research assistant and assessor) working at the study site provided the usual care to the control group that was monitored by the research assistant.

### 2.3. Task Selection

For reach-to-grasp (RTG) training, “drinking” task was selected because it is a common, goal-directed, meaningful functional task of real world [14], being the most trained task in many studies [4, 6–8, 36], and was a task of participants' choice while testing parameters of this study. Participants preferred three varieties of vessels (glass, bottle, and cup with handle; all regular size, regular type, common everywhere, and used in daily life) for drinking during parameter testing. Therefore, each participant was asked to choose one of the vessels while training in the drinking task (patient-therapist collaboration) [22].

### 2.4. Interventions

A single session of action-observation-execution (AOE) with accelerated skill acquisition program (ASAP) was administered. Both groups received AOE at first. Then, the experimental group received ASAP whereas control group received usual care.

#### 2.4.1. Action-Observation-Execution

**Figure 2:**
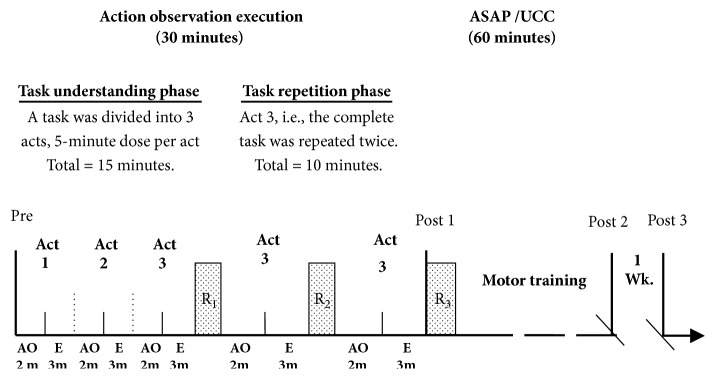
Intervention structure with timeline diagram of action-observation-execution. AO: action-observation, E: execution, ASAP: accelerated skill acquisition program, UCC: usual and customary care, m: minutes, R1 and R2: rest time between repetitions = 5 minutes, R3: rest time between interventions = 10 minutes, Wk.: week. Act 1: reach and grasp a glass/bottle/cup with the affected hand; return to the starting point. Act 2: reach, grasp, and raise a glass/bottle/cup toward the mouth with the affected hand; return to the starting point. Act 3: reach, grasp, and raise a glass/bottle/cup toward the mouth with the affected hand and drink; return to the starting point.

The drinking task was divided into three motor acts in order of progression [6, 8] to reduce the complexity of the task, so-called task understanding phase. The complete task was practiced [38] at later phase, two times with rest in between [7, 16], so-called repetition phase (Figure 2), to make the imitation more effective [39].

For action-observation, videos were prepared from nondisabled individual [6, 8], as a first-person perspective [6, 29]. Each participant observed the video of his/her preferred task on a computer screen kept at one-meter distance in a quiet room [4, 13, 16]. Participants were consistently instructed at the beginning of each video to observe with intention-to-imitate [6, 8, 16] and to silently count the number of repetitions of each video to enhance attention [13, 25]. Stefan K et al. [13] found that participants' alertness and attention to the task were not found different even on asking to count the number of odd movements. Rather, it enhanced attention. After AO, participants had to execute the observed action. Execution was enhanced applying the principles like “use it and improve it”, “specificity”, “repetition”, “intensity”, and “salience” [38]. Motivation was enhanced using ASAP [21]. Though a wide variation of dose of AO (1 to 6 minutes) and practice (1 to 6 minutes) is evident [4, 6–8, 13–15, 40], we administered a dose of 2-minute AO (long enough but sustained attention) and 3-minute execution (long enough but no fatigue) per act (total 30 minutes with rest time) as per the conclusion obtained during parameter testing.

#### 2.4.2. Accelerated Skill Acquisition Program

As per our objectives, we modified task selection strategy (described above) from the one described by Weinstein CJ et al. [22, 41]. Participants were well oriented regarding their collaborative role and active participation. The task as well as movement analysis was carried out to determine key impairments and movement breakdown points. The impairments type, severity, and the structures involved were identified. Each participant was encouraged for self-assessment to explore the problem lists and limiting factors. The key impairments limiting drinking function were identified and treated. Compensatory movements were avoided. The empowerment was embedded in the training. Performance threshold was identified for training and progression. The training was interactive. Success was celebrated to build up confidence. The ASAP's operating-principles [21] and classic exercise-overload-principles [21, 38] were applied for building motor capacity and progression. Participants took rest when required but kept to a minimum. The steps for integration of ASAP elements and administration procedure were based on Winstein CJ et al. studies [21, 22, 41]. The ASAP was administered for 60 minutes in addition to AOE (Figure 2).

#### 2.4.3. Dose-Equivalent Usual and Customary Care (DE-UCC)

The intervention being used in daily practice to treat stroke survivors with the characteristics similar to the participants of this study was termed UCC. We received UCC protocol from each physiotherapist of the site in advance. It consisted of active, active-assisted, and passive movements, neurodevelopmental techniques, stretching, strengthening, and coordination exercises. The participants in control group received UCC of 60 minutes, hence termed DE-UCC.

### 2.5. Outcome Measurement

Since our objective was to measure the immediate effect of a single session of the intervention focused on hand functions, the movement time (MT) and functional ability (FA) of individual as well as total of only six unilateral hand items and grip strength of the Wolf Motor Function Test (WMFT) were measured. The WMFT was a primary outcome measure because it is a valid, reliable, sensitive, and appropriate measure to detect intervention-induced behavioral changes particularly in stroke with mild-to-moderate severity [42–44]. It could also detect the immediate effect of single session of task-specific training [36, 45]. It was cross-culturally translated into Nepali language. Good to excellent criterion validity and reliability of the translated version were established [46].

The global recovery and hand functions were measured using stroke impact scale (SIS) [47]. The intrinsic motivation was measured using stroke rehabilitation motivation scale (SRMS) in the form of VAS [48]. The reliability and validity of these secondary outcome measures were established in patients with stroke and have been used as outcome measures [21, 28, 48, 49]. The self-efficacy was measured using ASAP brief self-efficacy rating scale that was developed particularly to assess self-efficacy in ASAP intervention [41, 50].

### 2.6. Statistical Analysis

We calculated sample size with reference to the study by Ertelt D et al. [16] to detect an effect size of 0.8 using n = 2*σ*_p_^2^ (Z*α*/2 + Z*β*)^2^ / (*μ*1 –*μ*2)^2^ at *α* = 0.05 and *β* = 0.02 where *σ*_p_^2^ represents pooled variance [51]. With 20% dropout, the sample size was found to be 12.

We described demographic data using mean (standard deviation) for the continuous, cumulative frequency for the categorical and median (interquartile range) for ordinal variables.

The Shapiro–Wilk test was used to examine the distribution of the data. Data transformation was done for parametric (continuous) variables showing nonnormal distribution. Greenhouse-Geisser correction was applied when sphericity was not achieved (Mauchly's test).

We used paired* t*-test and Wilcoxon-signed-rank test to compare parametric (normal distribution of continuous variables) and nonparametric (nonnormal distribution of continuous variables) data within groups, respectively. Two-factor mixed model ANOVA, repeated on time, was used to analyze main effects and group-by-time interaction for parametric data. Friedman's two-way ANOVA in conjunction with Mann–Whitney* U* test (for between-groups) was used to analyze nonparametric data.

Post hoc analysis was performed across and between tests with Bonferroni adjustments. For detecting the effect size (ES), the Cohen's d was calculated using degree of freedom and ratios of F- or t-statistics depending upon the test [52]. The p-value < 0.05 was considered significant. Analysis was carried out using SPSS 19.0 (Armonk, NY: IBM).

## 3. Results

Twelve right-handed eligible participants were enrolled (Table 1). There were no significant differences between groups at pretest in age, stroke type, lesion location, chronicity, and outcome variables as well as in any tools that were included in the selection criteria, which measured impairment level (p > 0.05). The MT was significantly reduced after AOE in flipping cards (mean change = 2.34 seconds, p = 0.01, ES = 0.7).

A marked reduction was found in total MT (mean change = 5.33 seconds, p = 0.06, ES = 0.53). There were marked changes in median score of total FA (1.5), FA of lifting can (0.6), and lifting paper clip (0.5) items. A significant difference was found in SRMS, self-efficacy, SIS-total hand function, carrying heavy objects, and turning a doorknob after AOE (Table 2).

Significant within-group difference was found in total MT (mean change = 43.28 seconds, p = 0.008, ES = 0.7), lifting pencil (mean change = 3.06 seconds, p < 0.001, ES = 0.9), and grip strength (ES = 0.7) only in the experimental group. Significant between-group difference was found in SIS-global recovery (ES = 0.5). However, between-group difference in grip strength was not significant (p = 0.06). Significant group-by-time interaction was found (Table 3) in pencil lifting item (ES = 0.6), total MT (ES = 0.6), and SIS-global recovery (ES = 0.53). The simple effects for pencil lifting item was significant at post 2–pre (< 0.001), post 3–pre (< 0.001), and post 3–post 1(< 0.001); however an increased time at post 1 was due to an outlier; for total MT, it was significant at post 2–pre (p < 0.001), post 3–pre (p < 0.001), post 2–post 1(p = 0.004), and post 3–post 1(p < 0.001), and for SIS–global recovery, it was significant at post 2–pre (p = 0.004), post 3–pre (p < 0.001), post 2–post 1(p = 0.01), and post 3–post 1(p < 0.004). Furthermore, the SIS-global recovery level continuously and uniformly increased in the experimental group whereas it is dropped back in control group and we found significant between-groups differences at post 2 (p = 0.001) and post 3 (p < 0.001).

**Figure 3:**
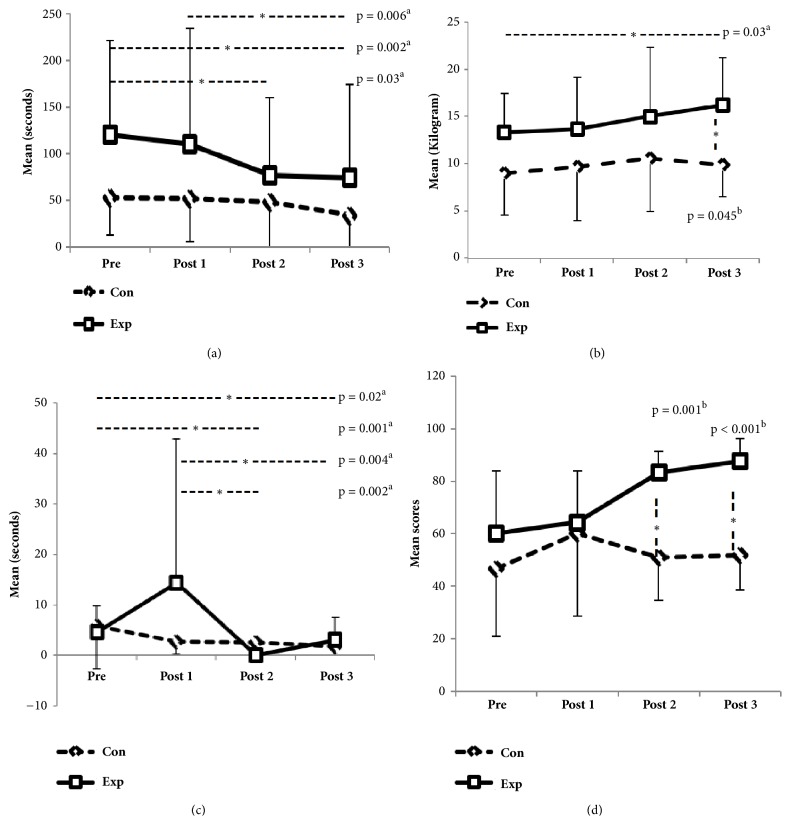
Graphs showing group-by-time interaction with pairwise comparisons for (a) Wolf motor function test-total movement time, (b) grip strength, (c) pencil lifting item, (d) stroke impact scale-global recovery; Con: control group, Exp: experimental group, *∗*: significant difference at p < 0.05, a: significant across the tests in the experimental group, b: significant between the groups. The data transformation has addressed the issue regarding the standard deviation that was larger than mean 14.44 (28.48) at post 1 for pencil lifting item.

The FA of WMFT items except flipping cards and total SIS hand functions as well as carrying heavy objects (p = 0.01), turning a doorknob (p = 0.03), and picking up a dime (p = 0.007) of SIS hand functions demonstrated significant differences within the experimental group. However, SRMS and self-efficacy showed significant differences across the tests in both groups (Table 4). Post hoc analysis (Figure 3, Table 4) revealed that most of variables, including subitems of SIS hand functions, showed significant difference between post 2–pre, post 3–pre, and post 3–post 1. There were no significant changes in any variables between post 3 and post 2.

## 4. Discussion

We investigated the immediate effect of AOE alone and in combination with ASAP in patients with subacute stroke. The immediate beneficial effect of AOE on UE functions was strengthened with integration of ASAP. The combined effect persisted for at least a week.

The participants were relatively heterogeneous on age, type of stroke, and lesion location. The dramatic spontaneous improvements might have already occurred at acute stage (within one month) [53] and exercise induced motor improvement significantly continues until six months [18, 53]. Therefore, the improvement seen at subacute stage (1-6 months) [54] could be dominantly intervention induced. Participants completed the study without any adverse effects. Since, the actual number of repetitions of the videos and the number that the participants counted were not significantly different, all participants had observed the videos with attention.

### 4.1. The Effect of AOE Integrated with Structured Motivation on UE Functions

The faster RTG actions after AOE with medium to large ES on MT of lifting can and flipping cards as well as total MT indicated contribution of AOE to the improvement of UE performance. The AOE-induced change (1.3 to 2.34 seconds) met the established minimal clinically important difference (MCID) of WMFT [44] that showed clinical importance of the intervention. Increased self-confidence and participants' interest supported the significant improvement in flipping cards. The reduction in movement time and improvement in functional ability of lifting can task demonstrated task-specific result. Significant improvement in global recovery, motivation, self-efficacy, and SIS hand functions further strengthened the result of primary variables. To the best of our knowledge, this was the first study to demonstrate immediate effect of one session of AOE integrated with motivation in patients at subacute stroke.

An outlier at post 1 in experimental group was found in lifting-pencil item. The participant who demonstrated an outlier has got his middle finger amputated at first interphalangeal joint due to trauma one year before he got stroke. When we assessed him, there was no pain. However, as per the case history, he used to get pain during some finger movements occasionally after amputation. The pain used to be neither specific to any movements nor specific to any fingers. Therefore, we did not exclude him from the study. Therefore, pain could be the reason in taking longer time for lifting pencil during only post 1 which yielded longer average time for the task. However, we could not delete the outlier because it would have distorted the matching and group allocation.

Our finding was consistent with the studies in acute [6–8] and chronic [15, 16] stroke. These studies investigated combined effect of 2-4 weeks of AOE (without any structured motivational component) and practice. We motivated participants based on ASAP thereby increased attention and engagement during observation and encouraged practice [22, 41]. We divided a task into three motor acts, first to make the movement easy and then to enhance imitation through repetition. These evidence-based strategies [20, 38] might have strengthened AOE to demonstrate beneficial effect with a single dose in present study.

Based on neuroimaging-based studies [13, 55] that demonstrated neuronal reorganization with AO-based interventions, AOE in this study might have enhanced exercise-dependent neural plasticity. Ertelt D et al. [16] demonstrated both behavioral changes and neuronal reorganization with AO and motor training. We can argue for the evidence that the improved UE functions with AOE in present study might be associated with activation of mirror neurons. The intact cortical regions in the majority of participants indicated that the core MNS (inferior frontal gyrus, ventral premotor cortex, inferior parietal lobule) [11] might have contributed to enhanced mirror neurons activity. Meanwhile, the cognitive control, motivation, and emotional responses might have enhanced the neural system (orbitofrontal cortex and amygdala) [23]. Thus, the mirror neurons in cortical areas might have contributed to motivational enhancement and increased neuronal activation to produce behavioral induced neural plasticity during AOE integrated with motivation.

Elisabetta S et al. [56] reviewed 20 randomized controlled trials (from or before 1982 to 2015) and found the beneficial effect of AO therapy on motor training including dexterity in subacute stroke. Kim K et al. [10] conducted another review of randomized controlled trials (from 2000 to 2014) in which all included studies demonstrated significant improvement in motor function with AO therapy except a study by Cowles T et al. [7], which did not show significant effect of AO therapy, which could be because they trained participants without videos. Incongruent movements between execution and observation also could not demonstrate better effect like that of congruent movements [25]. The administration variation might be the reason for showing little to no beneficial effect of AO therapy in some studies [7, 25].

### 4.2. The Combined Effect of AOE and ASAP on UE Functions

The significant group-by-time interactions and large-effect size suggested influence of intervention between groups across the tests. The reduction in MT and the consistent findings of secondary outcome variables indicated priming effect of AOE on ASAP. The clinically important changes (that met the MCID of WMFT: 1.54 to 1.6 seconds) [44] and larger effect size obtained across the tests only in experimental group indicated significant improvement of UE functions in participants receiving ASAP. The significant difference found within the group was only in experimental group. This explains that the effect of spontaneous recovery could have minimum effect. The motivation was integrated during AOE in both the groups, so significant differences within the group were seen in both groups. This was the first study, to the best of our knowledge, demonstrating priming effect of AOE on ASAP with sustainability effect in subacute stroke.

The improved performance could be due to two simultaneous memory processes on common neural substrate, which has been demonstrated by neuroimaging-based studies [16, 57]. The combined effect of action-observation and practice has been found to exceed the simple addition of their effects [13, 16, 55]. The priming effect of AOE on conventional physiotherapy has been demonstrated in patients with early [6–8] and chronic stroke [15, 16], which was in agreement with present findings in subacute stroke.

We found a beneficial effect of ASAP over DE-UCC. Tretriluxana J et al. demonstrated significant improvement on RTG and WMFT tasks [28]. Lump P S et al. demonstrated improved quality with faster movement in participants receiving ASAP compared with those receiving usual care [27]. Present findings were consistent with those two studies. Theoretically defensible and evidence-based ASAP model [22, 41] itself also supported present result. However, Winstein CJ et al. [50] did not find superiority of ASAP over usual care in performance based outcome measures, in contrast to our findings. In our understanding, the priming effect from AOE plus the beneficial effect of 90 minutes/session of our protocol, compared with only 60 minutes in their trial, could have led to the enhanced effect of ASAP in present study. Two hours per session of ASAP has already shown beneficial effect [27, 28]. The priming effect of AOE on conventional motor training is also well established [16, 57]. This evidence along with the concluding remark of the trial of Winstein CJ et al. (30 hours, if delivered in shorter time, the ASAP could be more effective) [50] favored the findings of the present study. Meanwhile, the Interdisciplinary Comprehensive Arm Rehabilitation Evaluation trial (publication by Lewthwaite R et al. 2018) [58] demonstrated enhancement in patient-reported outcomes (SIS), participants' confidence, and participation level with ASAP intervention, which is consistent with our findings. The improvement was achieved more quickly with the ASAP.

The AO induced activation on ventral premotor cortex, inferior parietal lobe, primary motor area, inferior frontal gyrus, and supplementary motor area [11] and the imitation induced activation in the superior temporal sulcus and parietofrontal mirror system [11, 13] improved UE performance. Moreover, the activity from multiple sensory inputs and corticospinal facilitation through MNS further enhanced the beneficial effect of the combination [11, 59]. The increased activation through mesocortical pathway [59] and the added benefits through the ingredients of ASAP [22] might have contributed to the functional improvement of UE. As per Lewthwaite R et al. 2018, strategies to support confidence building and therapist-patient collaboration throughout the session facilitate behavioral changes [58]. This could be the reason for enhanced motivation in this study.

### 4.3. Task Specificity Findings

The significant improvement on lifting pencil for both MT and FA indicated task specificity, which is consistent with previous studies [15, 36, 45]. Out of the lifting items, the relatively low values on lifting can and paper clip could be the impact of the challenge with the weight and size, respectively. Due to the absence of full flexion of interphalangeal joints of index finger, participants used to have pulp grasp between index finger and thumb to hold the cup, which is similar to that of grasping pencil to lift it up. Moreover, four of six participants of experimental group selected a cup with a handle as a training task. Therefore, lifting-pencil function might have improved due to task specificity.

The significant improvement found only in unilateral subitems of SIS hand function supported the result of MT of the WMFT. Our finding is also consistent with motor control and learning evidence, which has demonstrated that the transfer of skills from simple to complex tasks is more difficult. The more similar the tasks are, the better the transfer of skills is [60]. The protocol of present study allowed practicing drinking task by dividing it into three motor acts. This might have enhanced reaching and grasping actions. The transfer of skills with a single session of treatment might be less on the items that had different grasps than that of the trained task on which the performance improvement could be due to the contribution of the improved reaching action rather than grasping actions.

### 4.4. The Treatment Effect on Grip Strength

The improvement found on grip strength during retention test could be due to the contribution of the ASAP intervention. The result could be due to two beneficial effects of ASAP: (a) weakness was directly dealt with to build up capacity during the training, and (b) participants were motivated for building up of confidence, self- assessment, and self-management [22, 41] which might have encouraged them to apply skills in their daily activities and practice. ASAP provided favorable and meaningful engagement in activities to gain strength continuously. The celebration strategies in ASAP after gaining improvement might have further developed confidence through the activation of mesocortical pathway. Rather than just strengthening as in control group, weakness was addressed and participants of experimental group were encouraged to do the function. Confidence level was increased and active participation was promoted through celebration of the success. This might have helped them continuously engage in their functions [22]. Therefore, the grip strength significantly improved at retention test but could not show significant improvement immediately after intervention. The present finding was supported with the evidence that increase in strength immediately after functional training is similar to that of resistance training, but long-term strength gain is better with the functional training than that of resistance training [37].

### 4.5. Sustainability of the Benefit of AOE Combined with ASAP

The performance improvement achieved with the combination of ASAP and AOE sustained for at least a week. Though both groups continued their routine treatment or activities during one-week period after end of treatment, the sustainability of the improvement was seen only in the experimental group. This was consistent with the sustainability effect following 150 minutes of AO and practice in Lee D et al. [15] and 60 minutes of ASAP with brain stimulation in Thanakamchokchai J et al. [36]. The design and protocol of these two studies were comparable with the present study and the findings were consistent.

### 4.6. Study Limitations

We evaluated functional performance using behavioral measures. The neuroimaging measures would have provided additional information about intervention-induced neuronal reorganizations. The contribution from repeated measures could be a question of learning effect. It was unlikely because both groups were assessed for equal times by single assessor who was blinded to group allocation. A small sample size would limit its clinical application. Therefore, we recommend for large-scaled studies confirming the findings and exploring the long-term effect of the intervention in individuals with stroke.

## 5. Conclusions

The present study provided evidence that the beneficial effect of AOE got enhanced when integrated with structured motivation. The ASAP was primed with AOE and the combination elicited enhanced UE function that persisted for at least one week. These findings indicated the combined use of AOE and ASAP for dexterity training in subacute stroke.

## Figures and Tables

**Table 1:** Participants' characteristics and clinical data at the time of recruitment.

**Participants**	**Age (Years)**	**Gender**	**Type of stroke**	**Side of lesion**	**Lesion location**	**Days after stroke**	**FMA score**		**MMSE score**
							**UE** ^**a**^	**Hand** ^**b**^	
**Control group**									
1	40	Female	I	Left	Corona radiata	80	42	2	25
2	60	Male	I	Left	Thalamus	138	52	3	29
3	68	Female	H	Left	Thalamus	64	48	3	27
4	65	Female	I	Right	Subcortical	79	50	4	26
5	34	Male	I	Right	Basal ganglia	37	50	3	28
6	72	Female	I	Right	Thalamus	32	44	2	24
Mean (SD)	56.5 (15.72)					71.7 (38.4)	47.67 (3.89)	2.83 (0.75)	26.5 (1.87)
Frequency		2 Male/4 Female	5 I/1 H	3 Left/3 Right					
**Experimental group**									
7	63	Female	H	Left	Thalamus and parietal lobe	45	46	3	24
8	62	Male	I	Right	Cortical and sub-cortical	122	52	3	28
9	39	Male	H	Right	Basal ganglia	159	38	2	25
1	69	Male	I	Right	Thalamus	53	48	4	27
11	55	Male	H	Left	Thalamus	101	49	3	28
12	43	Male	I	Left	Caudate and corona radiata	39	48	4	29
Mean (SD)	55.17 (11.91)					86.5 (48.6)	46.83 (4.75)	3.17 (0.75)	26.83 (1.94)
Frequency		5 Male/1Female	3 I/3 H	3 Left/3 Right					

FMA: Fugl Meyer assessment, MMSE: mini-mental state examination (out of 30), UE^a^: motor and coordination score of upper extremity (out of 66), Hand^b^: flexion/extension score of FMA-hand item (out of 4), SD: standard deviation, I: infarction, H: hemorrhage.

**Table 2:** Comparison before and after action-observation-execution.

**Variables**	**n**	**Pre test** Mean (SD)	**Post test** Mean (SD)	**p-value** ^**a**^	**Pre test** Median (IQR)	**Post test** Median (IQR)	**p-value** ^**b**^
**WMFT-movement time **(second)					**WMFT-functional ability **(score)		
Total	12	86.35 (81.17)	81.02 (94.43)	0.06	18.5 (13.25, 21.75)	20 (13.25, 22.75)	0.25
Lift can	12	3.30 (4.55)	2.46 (2.02)	0.21	3.4 (3, 4)	4 (3, 4)	0.63
Lift pencil	12	5.24 (6.71)	8.59 (20.21)	0.13	3 (2.25, 4)	3 (2.25, 4)	1.00
Lift paper clip	12	2.51 (1.69)	2.36 (1.22)	0.70	3.5 (3, 4)	4 (3, 4)	0.25
Stack checkers	12	20.64 (32.26)	20.71 (32.62)	0.67	3 (2, 3.75)	3 (2, 4)	0.75
Flip cards	12	13.48 (5.52)	11.14 (5.02)	0.01*∗*	3 (2, 3.75)	3 (2, 4)	0.75
Turn key in lock	12	41.18 (47.68)	35.75 (43.41)	0.12	2 (1.25, 3)	2 (2, 3)	0.75
Grip strength (Kg)	12	11.12 (4.06)	11.65 (3.87)	0.13			
**Stroke impact scale **(score)							
Global recovery	12	53.33 (24.71)	62.08 (25.18)	0.07			
Hand functions							
Total	12				10.5 (6.75, 14.75)	13.5 (7.75, 18.75)	0.02*∗*
Carry heavy objects	12				1.5 (1, 3)	2.5 (1, 4)	0.02*∗*
Turn a door knob	12				2.5 (1, 3)	3 (2, 4)	0.03*∗*
Open a can or jar	12				2.5 (1, 3)	2.5 (1, 4)	0.25
Tie a shoe lace	12				1 (1, 2.75)	2 (1, 2.75)	0.63
Pick up a dime	12				2 (2, 3.75)	3.5 (2, 4)	0.08
**SRMS**–intrinsic motivation (score)	12				20 (16.25, 25)	26.5 (21.75, 27.75)	0.002*∗*
**Brief self-efficacy rating scale** (score)	12				7 (5, 8)	9.5 (9, 10)	0.001*∗*

SRMS: stroke rehabilitation motivation scale, p-value ^a^: from paired t-test, p-value ^b^: from Wilcoxon signed rank test. *∗* Significant at p < 0.05, n: number of participants, SD: standard deviation, IQR: interquartile range, WMFT: Wolf motor function test.

**Table 3:** Result of within and between group analysis on parametric data.

**Variables**	**Control group**					**Experimental group**					**p-value** ^**a**^	**p-value** ^**b**^
	**Pre**	**Post 1**	**Post 2**	**Post 3**	**p-value**	**Pre**	**Post 1**	**Post 2**	**Post 3**	**p-value**		
**Wolf motor function test**												
**Movement time **(seconds)												
Total	52.28 (39.98)	51.75 (45.95)	47.79 (29.84)	33.60 (12.72)	0.16	120.41 (100.56)	110.29 (124.29)	77.13 (82.61)	73.86 (99.90)	0.008*∗*	0.52	0.02*∗*
Lift can	4.63 (6.37)	2.93 (2.79)	3.09 (2.24)	2.71 (2.05)	0.20	1.96 (0.77)	2.00 (0.80)	1.55 (0.63)	1.29 (0.52)	0.17	0.20	0.29
Lift pencil	5.83 (8.44)	2.74 (2.38)	2.56 (1.21)	1.83 (0.37)	0.18	4.65 (5.21)	14.44 (28.48)	1.59 (0.54)	3.08 (4.44)	< 0.001*∗∗*	0.57	0.03*∗*
Lift paper clip	2.99 (2.32)	2.71 (1.64)	2.17 (0.95)	1.95 (0.53)	0.39	2.04 (0.58)	2.00 (0.54)	1.57 (0.42)	2.25 (1.38)	0.23	0.43	0.45
Stack checkers	9.97 (6.21)	11.99 (10.60)	15.13 (15.09)	9.79 (7.08)	0.71	31.30 (44.47)	29.44 (45.23)	15.54 (21.92)	25.69 (46.78)	0.06	0.88	0.12
Flip cards	12.67 (6.67)	10.68 (5.71)	11.16 (7.44)	7.92 (2.50)	0.13	14.28 (4.59)	11.61 (4.71)	11.07 (6.02)	10.35 (5.52)	0.17	0.50	0.75
Turn key in lock	16.19 (11.89)	20.69 (23.90)	13.70 (6.68)	9.40 (3.82)	0.41	66.18 (57.97)	50.81 (55.10)	45.81 (58.34)	31.20 (45.07)	0.08	0.52	0.24
**Grip strength **(Kilogram)	8.95 (2.96)	9.67 (3.07)	10.58 (3.37)	9.87 (5.20)	0.11	13.28 (4.02)	13.63 (3.75)	14.97 (4.69)	16.17 (4.29)	0.02*∗*	0.06	0.13
**Stroke impact scale **(score)												
Global recovery	46.67 (25.82)	60.00 (31.62)	50.83 (16.25)	51.67 (13.29)	0.18	60.00 (23.87)	64.17 (19.60)	83.33 (8.16)	87.50 (8.80)	0.14	0.04*∗*	0.03*∗*

p-value ^a^: between-groups main effect from ANOVA, p-value ^b^: two-way mixed ANOVA repeated on time (group *∗* time) on transformed data (log transformation for can lifting and paper clip lifting items, reciprocal transformation for pencil lifting, checkers stacking, cards flipping and turning key in lock items, and 1/square root transformation for WMFT-total movement time) [52]. Same transformation has been applied to all the variables that have to be analyzed together. Different transformations were selected for different items to fit the transformation technique to each variable of that item [52]. *∗*p < 0.05, *∗∗*p < 0.001; values across tests are given as mean (standard deviation) unless otherwise indicated.

**Table 4:** Result of within group analysis on nonparametric data.

**Variables**	**Control group**					**Experimental group**				
	**Pre**	**Post 1**	**Post 2**	**Post 3**	**p-value**	**Pre**	**Post 1**	**Post 2**	**Post 3**	**p-value**
**Wolf motor function test-functional ability (scores)**										
Total	19.5 (13.5, 23.0)	21.5 (13.75, 23.0)	19.5 (15.0, 25.0)	20.5 (17.5, 23.75)	0.29	18.5 (13.0, 20.5)	18.0 ^a^ (12.75, 23.75)	24.5 ^b, c^ (18.75, 26.5)	24.5 ^d. e, f^ (18.25, 28.25)	< 0.001*∗∗*
Lift can	3.5 (2.75, 4.0)	4.0 (2.75, 4.25)	3.5 (2.75, 4.25)	3.5 (2.75, 4.0)	0.76	3.5 (3.0, 4.25)	3.5 (3.0, 4.25)	4.0 (4.0, 5.0)	5.0 (3.75, 5.0)	0.01*∗*
Lift pencil	3.0 (2.75, 4.0)	3.0 (2.75, 4.25)	3.5 (2.75, 4.25)	3.5 (3.0, 4.25)	0.53	3.5 (2.0, 4.0)	3.0 (2.0, 4.25)	4.5 ^b^ (4.0, 5.0)	5.0 ^d, e^ (3.25, 5.0)	0.009*∗*
Lift paper clip	4.0 (2.0, 4.25)	4.0 (2.75, 4.25)	4.0 (3.0, 4.25)	4.0 (3.0, 4.25)	0.81	3.0 (3.0, 4.0)	3.5 (3.0, 4.25)	4.0 ^b^ (4.0, 4.25)	4.00 (3.0, 5.0)	0.01*∗*
Stack checkers	2.5 (2.0, 4.0)	4.0 (2.0, 4.0)	2.0 (2.0, 4.0)	3.5 (2.0, 4.0)	0.25	3.0 (1.75, 3.25)	2.5 (1.75, 3.25)	3.5 (2.75, 4.25)	3.5 (2.5, 5.0)	0.007*∗*
Flip cards	2.5 (2.0, 4.0)	3.5 (2.0, 4.0)	3.5 (2.0, 4.0)	3.5 (2.75, 4.25)	0.64	3.0 (2.75, 3.25)	3.0 (2.0, 4.0)	3.5 (2.75, 4.0)	3.5 (3.0, 4.0)	0.16
Turn key in lock	3.0 (2.0, 3.25)	2.5 (2.0, 3.0)	3.0 (2.75, 4.0)	3.5 (2.75, 4.0)	0.05	1.5 (1.0, 2.25)	2.0 (1.0, 4.0)	4.0 (1.75, 4.25)	4.0 ^d, e^ (2.75, 5.0)	< 0.001*∗∗*
**SIS**- total hand function score	8.5 (5.75, 14.25)	11.5 (6, 18.25)	12.5 (7.5, 17.25)	12.5 (11.5, 18)	0.12	10.5 (8.25, 15.5)	14 ^a^ (10, 20.75)	17.5 ^c^ (12, 24)	18.5 ^d, e^ 14, 20.5)	0.001*∗*
**SRMS- **intrinsic motivation score	19.0 (16.5, 25.25)	25.0 ^b^ (20.75, 30.0)	30.0 (25.5, 30.0)	25.0 (22.25, 27.0)	0.001*∗*	21.5 (15.25, 25.5)	27.0 (24.75, 27.25)	28.5 ^b^ (25.25, 29.0)	28.5 ^d, e^ (24.75, 30.0)	0.001*∗*
**ASAP- **self-efficacy score	6.5 (1.75, 9.25)	10.0 (8.75, 10.0)	10.0 (9.75, 10.0)	9.0 ^d^ (8.0, 10.0)	0.009*∗*	7.0 (5.0, 8.0)	9.0 ^a^ (9.0, 10.0)	9.5 ^b^ (9.0, 10.0)	10.0 ^d^ (9.75, 10.0)	< 0.001*∗∗*

SIS: stroke impact scale, SRMS: stroke rehabilitation motivation scale, ASAP: accelerated skill acquisition program. The data in the table were analyzed using Friedman's two-way ANOVA. The pair showed significant difference if the difference of mean rank ≥ critical difference (3.17) which was calculated using an equation; Z_*α*  /k  (k+1)_ √ [k (k+1)/6n] [52], k: number of comparisons, n: total sample, *α*: 0.05, a: post 1–pre, b: post 2–pre, c: post 2–post 1, d: post 3–pre, e: post 3–post 1, f: post 3–post 2, *∗*p < 0.05, *∗∗*p < 0.001. Values in the table are given as median (interquartile range) unless otherwise indicated.

## Data Availability

Data are available on request from the primary and corresponding authors.

## Abbreviations

ANOVA: Analysis of variance
AO: Action-observation
AOE: Action-observation-execution
ASAP: Accelerated skill acquisition program
DE-UCC: Dose-equivalent usual and customary care
ES: Effect size
FA: Functional ability
FMA: Fugl-Meyer assessment
MT: Movement time
NIHSS: National Institutes of Health Stroke Scale
SIS: Stroke impact scale
SRMS: Stroke rehabilitation motivation scale
UE: Upper extremity
WMFT: Wolf motor function test.
